# Mesenchymal Stem Cells-Derived Exosomes as Dexamethasone Delivery Vehicles for Autoimmune Hepatitis Therapy

**DOI:** 10.3389/fbioe.2021.650376

**Published:** 2021-03-30

**Authors:** Jiawei Zhao, Yue Li, Rongrong Jia, Jinghui Wang, Min Shi, Yugang Wang

**Affiliations:** ^1^Department of Gastroenterology, Tongren Hospital, Shanghai Jiao Tong University School of Medicine, Shanghai, China; ^2^School of Medicine, Jiangsu University, Zhenjiang, China; ^3^School of Medicine, Shanghai Jiao Tong University, Shanghai, China

**Keywords:** mesenchymal stem cells, exosomes, dexamethasone, autoimmune hepatitis, delivery

## Abstract

Exosomes (Exos) are nanosized vesicles (around 100 nm) that recently serve as a promising drug carrier with high biocompatibility and low immunogenicity. Previous studies showed that Exos secreted from mesenchymal stem cells (MSCs) provide protection for concanavalin A (Con A)-induced liver injury. In this study, the protective effect of Exos is confirmed, and dexamethasone (DEX)-incorporated Exos named Exo@DEX are prepared. It is then investigated whether Exo@DEX can function more efficiently compared to free drugs and naive Exos in a Con A-induced autoimmune hepatitis (AIH) mouse model. The results show that Exo@DEX efficiently improves the accumulation of DEX in AIH in the liver. These data suggest that Exo@DEX is a promising drug carrier for AIH and could have applications in other diseases.

## Introduction

Autoimmune hepatitis (AIH) is characterized by a mild increase in serum transaminase concurrent with increased immunoglobulin G and circulating autoantibodies (Strassburg, 2010). Currently, the first-line therapy for AIH is prednisone combined with azathioprine. However, high doses of steroids could easily spread into the whole body and contribute to AIH patients abandoning such treatment due to drug complications (such as Cushing’s syndrome, hypertension, peptic ulcer, infection, and neurological symptoms) (Violatto et al., 2019). Furthermore, AIH could easily be exacerbated into liver fibrosis or even cirrhosis of the liver if the disease is badly controlled and that is when liver transplantation becomes a necessity. An alternative could be the drug “navigating” destination, the injured liver, and the systematic side effects could be extensively reduced (Carbone and Neuberger, 2013). Nowadays, tissue-targeted therapies are achieved through several drug delivery systems, including nanosphere (Liang et al., 2018), liposomes (Bartneck et al., 2015), mesoporous silicon (Chen et al., 2019), or gel (Wang et al., 2019), which are engineered to concentrate the drug at a specific tissue site. However, some artificially synthetic nanoparticles are still facing some problems, such as poor biocompatibility and biotoxicity.

Exosomes (Exos) are bilayer lipid vesicles naturally secreted by cells with a diameter of around 100 nm. At first, Exos were thought to be cell excretions and have no function, but several years later, they were found to be important materials for cell-to-cell communication (Bobrie et al., 2012; Tkach and Thery, 2016). In the last few decades, Exos have begun to hold great promise as a drug delivery vehicle for small-molecule drugs, nucleic acids, and specific proteins due to their high biocompatibility, low toxicity, and various biofunctions according to their parental cells. It was worth noting that, reportedly, Exos would normally accumulate in the liver because of the preferential liver uptake, which is extremely beneficial for the treatment of liver diseases (Borrelli et al., 2018; Gudbergsson et al., 2019). All these unique characteristics make Exos a promising drug delivery vehicle for AIH treatment.

Mesenchymal stem cells (MSCs) have gained much attention in recent years due to their therapeutic effects in various diseases. These cells are called pluripotent stem cells that can differentiate into all kinds of functional cells under certain stimulation or conditions (Borrelli et al., 2018). It has been reported that resveratrol-treated MSCs can secret platelet (PLT)-derived growth factor-DD, which activates the signal-regulated kinase pathway in renal tubular cells and promotes angiogenesis in endothelial cells to repair the cisplatin-induced kidney injury (Zhang et al., 2018). When it comes to liver diseases, MSCs exert a multifunctional effect on liver regeneration by inhibiting hepatocyte apoptosis, reversing liver fibrosis, and reducing inflammation (Alfaifi et al., 2018). Furthermore, Exos derived from MSCs can inherit their therapeutic functions. For example, Exos derived from placenta MSCs could increase utrophin expression and decrease inflammation and fibrosis in Duchenne muscular dystrophy (Bier et al., 2018). Moreover, Exos from MSCs can suppress concanavalin A (Con A)-induced liver injury (Tamura et al., 2016) and accelerate the recovery of hindlimb ischemia by activating VEGF receptors (Gangadaran et al., 2017).

Based on the above, we developed a liver-targeting Exo@DEX delivery system by loading dexamethasone (DEX) into MSC-derived Exos for the treatment of Con A-induced AIH (Bartneck et al., 2015). Our expectation is to combine the therapeutic features of MSC-Exos with the potent anti-inflammation drug DEX to achieve a synergistic treatment toward AIH, hopefully along with other liver diseases. More than that, our work also implies the application of Exos as a promising drug carrier.

## Materials and Methods

### Cell Culture

Mesenchymal stem cells were extracted and purified from mouse bone marrow-derived all-nucleated cells. Non-adherent cells were removed after 72 h, and attached cells were maintained for 16 days in α-Minimum Essential Media (α-MEM, Gibco) supplemented with 2 mM L-glutamine and 55 μM 2-mercaptoethanol. The human liver cell line L02 was cultured in Roswell Park Memorial Institute 1640 media (RPMI1640, Gibco), and the murine macrophage cell line RAW264.7 was cultured in Dulbecco’s modified Eagle’s medium (DMEM, Gibco). All cells were cultured in media containing 10% fetal bovine serum (FBS, Gibco), (1%, v/v) penicillin, and (1%, v/v) streptomycin. Cell cultures were incubated in a 5% CO_2_ incubator at 37°C.

### Isolation and Purification of MSC-Exos

Mesenchymal stem cell-Exos were purified from a cell culture supernatant by ultracentrifugation as reported before (Théry et al., 2006). Briefly, MSC culture medium was collected when the cells were 80–90% confluent. The supernatant was centrifugated at 300 × *g* and 2,000 × *g* for 10 min to remove cells and dead cells, respectively. The supernatant was then centrifugated at 10,000 × *g* for 30 min to eliminate cell debris. The final supernatant is then ultracentrifuged at 100,000 × *g* for 1 h twice to pellet the Exos. The pellet was resuspended in a convenient volume of phosphate-buffered saline (PBS). Isolated Exos were either kept at 4°C for up to a week or stored at −80°C for further use.

### Characterization of MSC-Exos

The expressions of CD47, HSP70, CD81, and TSG101 (Abcam) were evaluated by ProteinSimple Wes^TM^ Capillary Western Blot analyzer (PS-MK15, ProteinSimple). Antibodies were diluted in an antibody diluent with a 1:50 or 1:100 dilution (ProteinSimple kit). The morphology of Exos was identified by transmission electron microscopy (TEM) (Hitachi HT7700, Tokyo, Japan). The size distribution and zeta potential of Exos were determined by dynamic light scattering (DLS) and nanoparticle tracking analysis (NTA). Total protein of Exos was quantified using the bicinchoninic acid (BCA) assay kit (Thermo Fisher).

### Drug Encapsulation

Dexamethasone was encapsuled in Exos using a sonication method reported before (Wan et al., 2017). Briefly, 50 μg of DEX was mixed with 50 μg of Exos at a 10 mL total volume at room temperature for 30 min followed by sonication in a water bath sonicator (KQ-300DE) with the following settings: 40% amplitude, 3 cycles of 15 s on/off for 1.5 min with a 2-min cooling period between each cycle. After sonication, the mixture was incubated at 37°C for 1 h and further ultra-filtrated three times to remove excess free drugs.

For the analysis of the amount of DEX incorporated in Exos, high-performance liquid chromatography (HPLC) was carried out (Agilent 1200). Separation was performed with a C18 column (150 × 4.6 mm, Phenomenex, United States). The elution solvents were water containing 0.05% acetic acid (phase A) and acetonitrile (phase B). The absorbance wavelength was set at 240 nm.

### Internalization of MSC-Exos *in vitro*

Exosomes were labeled with 1,1’-dioctadecyl-3,3,3’,3’-tetramethylindodicarbocyanine perchlorate (DiD, Fanbo Biochemicals) according to the manufacturer’s instructions, and the excess free dye was removed with Exosome Spin Columns (Thermo Fisher). L02 and RAW264.7 cells were incubated with labeled Exos for 1, 2, or 4 h. For fluorescence microscopy, the cells were fixed with 4% paraformaldehyde at room temperature for 30 min. The cell membrane was stained with Alexa Fluor 488 phalloidin (Thermo Fisher), and the cell nucleus was stained with 4’,6-diamidino-2-phenylindole (DAPI, Solarbio). Fluorescence microscopy images were obtained by confocal laser scanning microscopy (CLSM, Nikon A1R). For flow cytometry, the cells were trypsinized, washed three times, and further resuspended in PBS. The fluorescence intensity of 10,000 cells was recorded by CytoFLEX LX (Backman, United States) and analyzed by FlowJo.

### Cell Proliferation Assay

L02 cells and RAW264.7 cells were seeded into a 96-well cell culture plate at a density of 10,000 cells per well for 24 h before any treatment. Afterward, the cell culture media were replaced with fresh ones containing DEX or Exo@DEX with an equivalent DEX dose at different concentrations (1, 5, and 10 μg/mL). After 24 h of incubation, the cell culture media were replaced with CCK-8 (Solarbio) solutions, and the cells were incubated for another 1 h in an incubator. The absorbance of each well was measured by a microplate reader (Infinite M200, Tecan) at 450 nm. The viability of L02 and RAW264.7 cells was then calculated according to the instructions.

For cell apoptosis experiments, L02 cells and RAW264.7 cells were seeded into a 24-well cell culture plate at a density of 25,000 cells per well for 24 h before any treatment. Afterward, the cell culture media were changed with Exo-free media or media containing DEX or Exo@DEX with an equivalent DEX dose at 10 μg/mL. After 24 h of incubation, cells were trypsinized and washed three times. Finally, the cells were resuspended in 1 mL binding buffer and stained in the dark by 5 μL annexin V–fluorescein isothiocyanate (FITC) and propidium iodide (PI) for 10 and 5 min, respectively, according to the manufacturers’ instructions. Cell apoptosis was immediately examined by flow cytometry.

### Supernatant Cytokine Detection

RAW264.7 cells were stimulated by lipopolysaccharide (LPS) (100 ng/mL) for 24 h to obtain pro-inflammatory macrophages and subsequently underwent thorough washing to remove LPS. Then, fresh media containing DEX (10 μg/mL), Exo (100 μg/mL), or Exo@DEX (100 μg/mL) were added. After 24 h of incubation, the cell supernatant was collected, and the cytokines (TNF-α, IFN-γ, and IL-1β) of the supernatant were detected by enzyme-linked immunosorbent assay (ELISA) kits.

### Animal Models

Six- to 8-week-old BALB/c mice were purchased from Beijing Vital River Laboratory Animal Technology Co., Ltd. Mice were acclimated for 7 days before any treatment. For the construction of an AIH mice model, Con A (Solarbio) was intravenously injected via the tail vein at a dose of 20 mg/kg of body weight. Exo (5 mg/kg), DEX (0.5 mg/kg), Exo@DEX (5 mg/kg), and control solutions were injected intravenously in a total volume of 100 μL right after Con A injection.

### *In vivo* and *ex vivo* Distribution of MSC-Exos

Exosomes were labeled with 1,1’-dioctadecyl-3,3,3’,3’-tetra methylindotricarbocyanine iodide (DiR, Fanbo Biochemicals) according to the manufacturer’s instructions, and the excess free dye was removed with Exosome Spin Columns. BALB/c mice (*n* = 3) were injected with 100 μL of DiR-labeled Exos through the tail vein. The fluorescence images of the mouse body were recorded by the KODAK *in vivo* imaging system FX Pro (KODAK, United States) at different time points.

For *ex vivo* imaging, mice were treated with DiD (Fanbo Biochemicals)-labeled Exos and were sacrificed at 2 h after the injection. The liver, kidneys, spleen, lung, and heart were collected and scanned for *ex vivo* imaging. Particularly, the livers were either grinded into single-cell suspension and filtered using a 60-μm mesh for flow cytometry or fixed with 4% paraformaldehyde for frozen sections. Frozen liver tissues were cut into 10-μm sections and further incubated with F4/80 antibody (Abcam). F4/80-positive cells were stained with FITC, and cell nuclei were stained with DAPI. Fluorescence images were obtained by CLSM.

For the analysis of the amount of DEX delivered to the liver, HPLC was carried out. All samples were treated as reported before (Violatto et al., 2019). Briefly, livers were collected and homogenized with methanol and acetonitrile, stirred for 30 min. Then water was added, and homogenization was repeated. Samples were further centrifuged at 10,000 × *g* for 10 min at 4°C. The supernatant was further cleaned up with solid-phase extraction using Sep-Pak C18 1-cc Vac cartridges, conditioned before use with 1 mL of methanol.

### Liver Enzyme and Histology

Blood was collected 8 h after the injection to detect serum alanine aminotransferase (ALT) and aspartate aminotransferase (AST) levels. Livers were also collected, fixed in 4% paraformaldehyde, and embedded in paraffin for histological analysis.

### Liver Tissue Cytokine Measurement

Obtained liver tissues were cut into pieces and homogenated on ice with an ultrasonic probe in radioimmunoprecipitation (RIPA) buffer (Solarbio) with 1 mM protein inhibitor. Protein concentrations were measured using a BCA assay kit. Lysates were centrifugated at 10,000 × *g* for 5 min at 4°C according to the instructions, and the suspension was collected and stored in −80°C for further use. Before tests, a pre-experiment was conducted to optimize the concentration. ELISAs were conducted to measure the following cytokines according to the instructions (mouse TNF-α ELISA kit, mouse IFN-γ ELISA kit, mouse IL-1β ELISA kit, mouse IL-6 ELISA kit, mouse IL-12 ELISA kit, and mouse myeloperoxidase ELISA kit, Solarbio).

### Macrophage Apoptosis in the Liver

Obtained liver tissues were cut into pieces and grinded into single-cell suspension. Cells were primarily treated with red blood cell (RBC) lysing regents to remove RBCs. Then, the cells were labeled with F4/80 for gating of macrophages. Finally, the cells were resuspended in 1 mL of binding buffer and stained in the dark by 5 μL annexin V–FITC and PI for 10 and 5 min, respectively, according to the manufacturers’ instructions. Cell apoptosis was immediately examined by flow cytometry.

### Toxicity Assessment

Exo@DEX (5 mg/kg) or PBS was intravenously injected into AIH mice. Blood samples and organs were collected from the two groups 24 h after treatment. Hematoxylin-and-eosin (H&E) staining of each organ was conducted to observe the histological effects on the heart, liver, spleen, lungs, and kidneys of mice. Anticoagulant blood was used to perform a routine blood test. Untreated blood was put on a stand for 2 h and centrifugated at 4°C for 10 min to obtain the serum. The obtained serum was used to detect blood parameters using an automatic biochemical analyzer.

## Results

### Fabrication and Characterization of Exo@DEX

**FIGURE 1:**
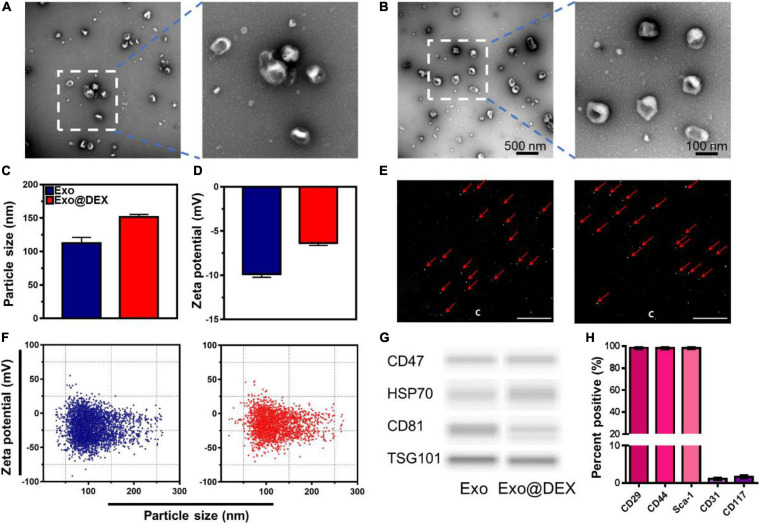
Characterization of Exo and Exo@DEX. **(A)** TEM imaging of Exos and **(B)** DEX-loaded Exos stained with uranyl acetate. **(C)** The size distribution of Exos and Exo@DEX by DLS. **(D)** The zeta potential of Exos and Exo@DEX by DLS. **(E)** Screenshots of Exo and Exo@DEX detection by NTA. **(F)** The size distribution and zeta potential of Exos and Exo@DEX by NTA. **(G)** Western blot analysis of Exos and Exo@DEX. CD47, HSP70, CD81, and TSG101 are markers for Exos. **(H)** Quantitative analysis of MSC markers of CD29, CD44, Sca-1, CD31, and CD117.

Exosomes were purified from cell culture supernatants of MSCs and were further loaded with DEX by water bath sonication. The morphology of both Exos and Exo@DEX was observed by TEM (Figures 1A,B). The size distribution and zeta potential of Exos and Exo@DEX were detected by both DLS and NTA. Naive Exos and DEX-loaded Exos both had a uniform size distribution, with a mean diameter of 120 and 150 nm, respectively (Figure 1C). The zeta potential of the two nanoparticles changed from around −10 to −6 mV due to the successful loading of DEX (Figure 1D). NTA showed a scattered distribution of the two nanoparticles during the detection progress. Movies and screenshots recording the movement of nanoparticles were provided (Figure 1E and Supplementary Movies 1, 2). The size distribution and zeta potential of both Exos were exhibited as scatter plots (Figure 1F). The size distribution was similar to that of DLS, while the zeta potential was more negative compared to the results of DLS, which may contribute to the different detection modes of the two equipment. However, the change of zeta potential was consistent with DLS. The Exo@DEX was then stored at −80°C for further use. The size and zeta potential did not have an obvious change (Supplementary Figure 1). Next, immunoblotting further verified that Exos and Exo@DEX expressed Exo markers (CD47, HSP70, CD81, and TSG101). It was shown that the drug loading did not alter the surface proteins of Exos (Figure 1G and Supplementary Figure 2). Importantly, we investigated the characteristics of the tenth-generation MSCs via flow cytometry. In brief, the expression of CD29, Sca-1, and CD44, positive markers of MSCs, was detected to be higher than 95%. On the other hand, the expression of the negative surface markers, CD31 and CD117, was determined by flow cytometry to be lower than 5% (Figure 1H and Supplementary Figure 3). The amount of DEX incorporated into Exos was measured by HPLC with the calculated DEX incorporation rate to be around 10% (Supplementary Figure 4).

### Cellular Internalization of Exo@DEX and Its Effect on Macrophage *in vitro*

The liver plays an essential role in human life. It is mostly composed of hepatocytes, around 80%, and other non-parenchymal cells, including hepatic stellate cells, Kupffer cells, and sinusoidal endothelial cells. Kupffer cells originate from macrophages and are involved in phagocytosis. In addition, Kupffer cells are fundamental in the process of innate immune and inflammatory responses. They can secrete several cytokines during the occurrence of inflammation and may even contribute to the acceleration of diseases. Thus, inhibition of macrophages could be considered as an underlying possibility to ameliorate the symptoms of inflammation activity. Steroids are the currently widely used therapy for the treatment of AIH in clinical settings. However, most patients may not be able to endure the severe side effects that steroid drugs bring. As a result, novel methods need to be explored to improve the targeting ability of drugs toward livers.

**FIGURE 2:**
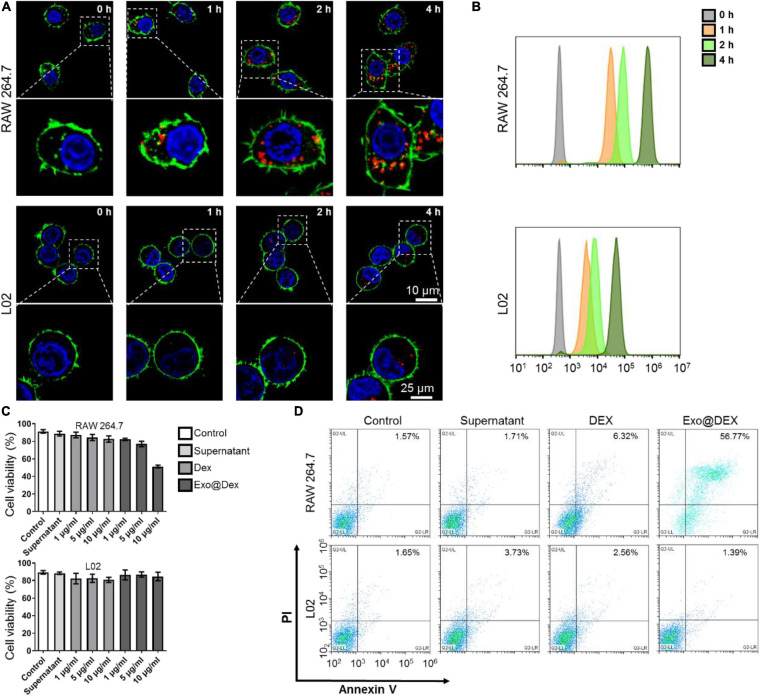
Cellular internalization of Exo@DEX and its effect on macrophage *in vitro*. **(A)** Representative fluorescence images of Exo uptake by RAW264.7 macrophages and L02 liver cells *in vitro* in a time-dependent way (images were enlarged, indicated by a white dotted box). Nuclei were stained with DAPI (blue), the cell membrane was stained with phalloidin (green), and Exo@DEX was stained with DiD (red). **(B)** Flow cytometry assay of RAW264.7 and L02 cells incubated with DiD-labeled Exo@DEX. **(C)** CCK-8 analysis of RAW264.7 and L02 cells. Cells were incubated with Exo-free supernatant (Supernatant), non-exosomal DEX, or exosomal DEX (Exo@DEX) for 24 h. **(D)** Flow cytometry analysis of annexin V-and-PI staining of RAW264.7 and L02 cells incubated with Supernatant, DEX, or Exo@DEX.

We used CLSM to test the effects of co-culturing Exo@DEX with two different types of cells *in vitro* and whether Exos can be internalized into these cells. We co-cultured Exo@DEX with macrophage cell line RAW264.7 cells and liver cell line L02 cells. After 0, 1, 2, and 4 h of co-incubation, a large number of Exo@DEX emitting red fluorescence were internalized into RAW264.7 macrophages, while L02 liver cells only took up fewer Exo@DEX than macrophages (Figure 2A and Supplementary Figure 5). Cellular internalization was also measured via a flow cytometry. Fluorescence intensity of RAW 264.7 cells increased obviously in a time-dependent way and had a higher level for Exo@DEX than the uptake level by L02 cells (Figure 2B).

Furthermore, to test the drug effect on the two cell lines, cells were treated with both free DEX and the Exo@DEX in a dose-dependent way (0–10 μg/mL) for 24 h. A CCK-8 cell proliferation assay was performed to examine the cell viability of the two cell types. After 24 h of co-incubation, the cell viability of RAW 264.7 macrophages was suppressed at a concentration of 10 μg/mL of Exo@DEX, while that of L02 cells was barely influenced at any concentration (Figure 2C). Once DEX gets inside the cell and further combines with corticosteroid receptors in the cytoplasm and nucleus, a variety of inflammatory genes and cytokines would be dramatically suppressed. To explore the mechanism of cell death induced by DEX, an annexin V-and-PI staining was performed to detect the cell apoptosis level of both cells after 24 h of co-incubation through a flow cytometry. In accordance with the cell viability test, the Exo@DEX group showed higher early and late apoptosis levels than the free drug in an equivalent dose of DEX, while L02 cells were not apparently affected (Figure 2D), possibly due to a lower internalization amount. We also measured the production of inflammatory cytokines, including TNF-α, IFN-γ, and IL-1β in the culture supernatants. Treatments containing DEX, Exo, and Exo@DEX efficiently reduced the production of TNF-α, IFN-γ, and IL-1β (Supplementary Figure 6).

### Exo@DEX Concentrate in the Liver Parenchyma

**FIGURE 3:**
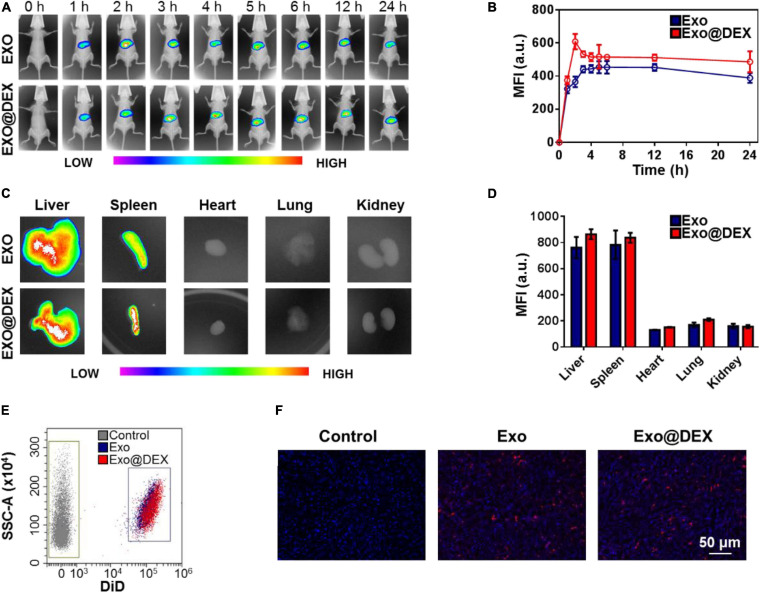
Biodistribution of Exo@DEX *in vivo* and *ex vivo*. **(A)** *In vivo* fluorescence signal of mice treated intravenously with DiR-labeled Exo or Exo@DEX and imaged 1, 2, 3, 4, 5, 6, 12, and 24 h after the treatment. **(B)** Quantitative analysis of *in vivo* imaging. **(C)** *Ex vivo* fluorescence signal of collected organs from mice sacrificed 2 h after treatment. **(D)** Quantitative analysis of *ex vivo* imaging. **(E)** Flow cytometry analysis of homogenated liver tissues after DiD-labeled Exo or Exo@DEX treatment. **(F)** Liver tissues excised 2 h after treatment with DiD-labeled Exo@DEX or PBS, stained with DAPI, and visualized by confocal microscopy.

One of the main purposes for the construction of Exo@DEX is to increase the accumulation of the small-molecule drug DEX in liver tissues. The biodistribution of Exo and Exo@DEX after intravenous (i.v.) administration was examined *in vivo* to detect their targeting ability. Exo and Exo@DEX were both labeled with DiR to allow tracking at different time points by an *in vivo* imaging system. The signal related to the DiR-labeled Exo and Exo@DEX was clearly observed through the imaging system and remained at a high level for 24 h (Figure 3A). Both Exo and Exo@DEX had a similar performance during a 24 h observation, indicating that the encapsulation did not affect the targeting ability of Exos. The fluorescence peak appeared at 2 h after i.v. administration (Figure 3B). *Ex vivo* imaging was performed 2 h after i.v. administration, and the fluorescence signal was detected in all collected organs (Figure 3C). The fluorescence signal was mainly discovered in the liver and spleen and was about three times higher than that in the heart, lung, and kidney (Figure 3D).

In addition, flow cytometry and frozen sections were used to further observe the distribution of Exos and Exo@DEX in livers. In these cases, DiR was replaced with DiD for a better imaging performance. Livers after i.v. administration were extracted and grinded into cell suspension for flow cytometry. As shown in Figure 3E, livers with DiD-labeled Exo and Exo@DEX treatment exhibited high fluorescence signals compared to Exos without any dye label. At the same time, collected liver tissues were preserved in 4% paraformaldehyde for a frozen section. DAPI staining was used to observe the nuclei, and a red signal represented the DiD-labeled Exo and Exo@DEX in liver tissues (Figure 3F). Furthermore, when hepatic macrophages were visualized by immunofluorescence staining of F4/80, most DiD-labeled Exo@DEX were localized with F4/80-positive cells (Supplementary Figure 7). For higher resolution, livers were extracted to analyze the amount of DEX delivered to them. Compared to free DEX, Exo@DEX was able to deliver about 2.6 times as much DEX to the liver (Supplementary Figure 8).

The data above were extremely important in terms of distribution because the drug-loaded Exos enable the accumulation of DEX in liver tissues and reduce free drugs in blood circulation. The underlying side effects of free DEX on unwanted organs therefore may not occur in the body of treated subjects.

### Exo@DEX Therapy Protects Liver Functions in Con A-Induced Liver Injury

**FIGURE 4:**
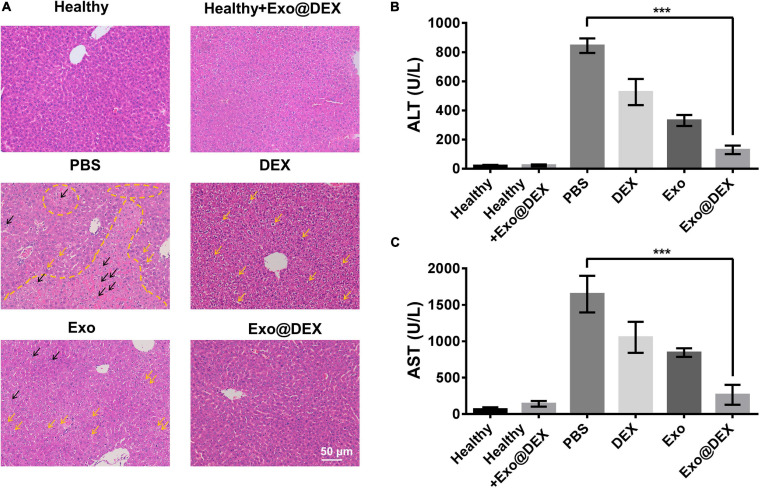
*In vivo* treatment of the Con A-induced AIH mouse model. **(A)** H&E staining of liver sections from different groups 8 h after Con A injection and treatment. Black arrows indicate inflammatory cells. Yellow arrows indicate steatosis, suggesting mild damage to livers. The yellow line indicates large hemorrhage, suggesting severe damage. **(B,C)** Serum levels of ALT and AST were measured 8 h after Con A injection and treatment (*n* = 5 for each group). Data in **B** and **C** are presented as the mean ± SD and were assessed via one-way ANOVA (***P < 0.001).

Hematoxylin-and-eosin tissue sections demonstrated that Exo@DEX therapy protected the structural integrity of the liver tissue. A healthy group with or without treatment showed no abnormality on the hepatic lobule and portal area, while obvious tissue structure disorders and severe cell necrosis could be seen in the PBS group, which was indicated by a yellow dashed line. Yellow and black arrows showed steatosis and inflammatory cell infiltration, respectively. DEX or Exo administration contributed to a certain extent to the protection of the liver structure and cell viability where mild damage indicated by steatosis could be seen. Exo@DEX treatment contributed to the recovery of tissue structure integrity, and few inflammation cells and little steatosis could be seen in this case (Figure 4A).

Alanine aminotransferase and AST are the two most used biomarkers for the analysis of liver functions. For the Con A-induced liver injury, the PBS group exhibited high ALT and AST levels. Compared to the PBS group, administration of DEX or Exo contributed to a certain degree to liver function protection. More importantly, injection of Exo@DEX resulted in a better therapeutic effect than the DEX or Exo group, mainly due to the targeting ability to the liver, suggesting the anti-inflammation role that Exo@DEX plays in Con A-induced liver injury (Figures 4B,C).

**FIGURE 5:**
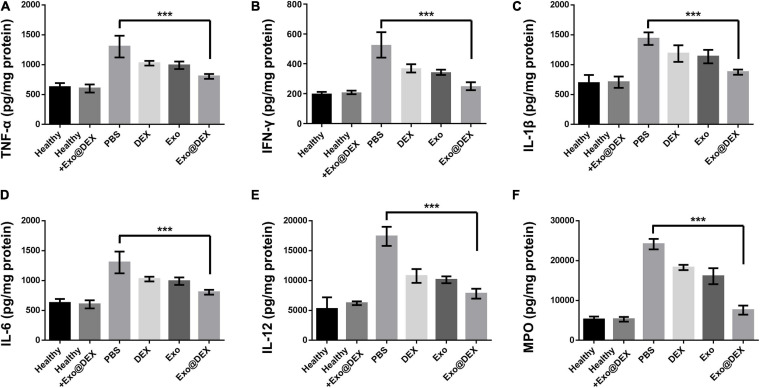
Detection of cytokines in liver tissues. Cytokines of TNF-α **(A)**, INF-γ **(B)**, IL-1β **(C)**, IL-6 **(D)**, IL-12 **(E)**, and MPO **(F)** were measured in liver homogenates form each group (*n* = 5 for each group). Data are presented as the mean ± SD and were assessed via one-way ANOVA (***P < 0.001).

Importantly, we grinded liver tissues into single-cell suspension and evaluated the apoptosis of macrophages. Macrophages from mice treated with Exo@DEX showed a higher apoptotic level than free drugs (Supplementary Figure 9). To further demonstrate the therapeutic effects of Exo@DEX, we detected several inflammatory cytokines in liver tissues from each group, including TNF-α, INF-γ, IL-1β, IL-6, IL-12, and MPO. As shown in Figure 5, the cytokine levels of Exo@DEX were dramatically decreased compared to the elevated cytokine levels of the PBS group (Figures 5A–F).

### Toxicity Study

**FIGURE 6:**
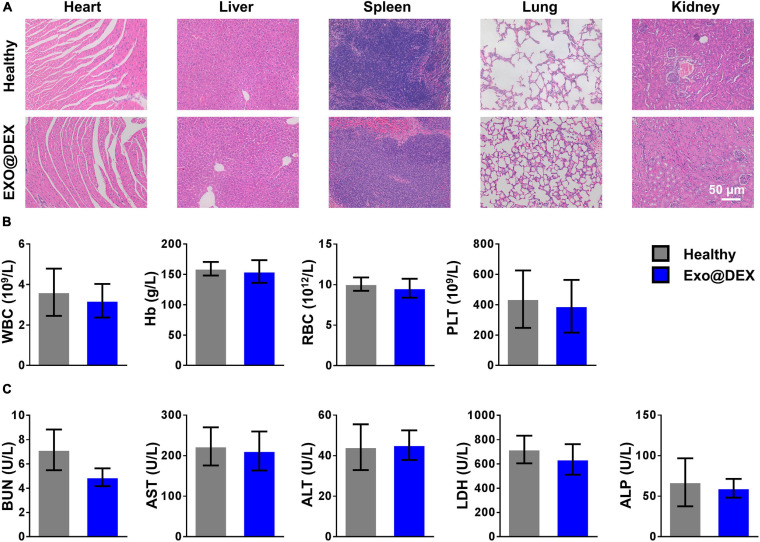
*In vivo* toxicity assessment of Exo@DEX. **(A)** H&E staining of major organs (heart, liver, spleen, lungs, and kidneys). **(B)** Routine blood test of whole-blood parameters in mice treated with PBS or Exo@DEX. **(C)** Serum levels of liver and kidney function parameters in mice treated with PBS or Exo@DEX.

In order to test the systematic toxicity that our treatment might have, healthy mice were given PBS or Exo@DEX to rule out potential side effects. All treatment did not contribute to any apparent signs of systematic toxicity. According to the H&E staining, no obvious tissue damage could be observed in the major organs (Figure 6A). We also confirmed that the above treatment did not cause any apparent hematologic abnormality because the levels of white blood cell (WBC), hemoglobin (Hb), RBC, and PLT were all within the normal range (Figure 6B). Meanwhile, levels of serum urea nitrogen (BUN) and ALT, AST, lactate dehydrogenase (LDH), and alkaline phosphatase (ALP), which are typical biomarkers for liver and kidney functions, were also examined, and no abnormality was observed (Figure 6C).

## Discussion

In this study, we constructed a drug-carrying Exo called Exo@DEX by loading the potent anti-inflammation drug DEX into MSC-Exos. The Exo@DEX exhibited effective protection against Con A-induced acute liver injury. There are advantages of the constructed nanoparticles. First, the size of Exo@DEX, around 100 nm, is bound to lead to long liver retention after tail vein injection. Second, the MSC-secreted Exos have preferable repair effects for injured tissues or organs. Third, the MSC-Exos possess great biocompatibility and low immunogenicity, making them good candidates as drug carriers. Fourth, DEX-loaded Exos can reduce the systemic bad effects on unwanted organs.

Exosome is a promising drug carrier with different properties according to its parental cells. As a vehicle, Exo has already been widely used in the treatment of cancers (Li and Nabet, 2019), including glioblastoma (Zhu et al., 2019), pancreatic cancer (Li et al., 2020), and breast cancer with lung metastasis (Xiong et al., 2019). All treatments exhibited effective inhibition in tumor volume and curative effect on the survival time of tumor-bearing mice. However, in the case of treatment for inflammatory diseases, limited research works have been done to explore the possibility for Exos to encapsulate other drugs other than chemotherapeutic drugs for cancer therapy. Our current study provides direct evidence that DEX-loaded Exos accumulate largely in the liver. Importantly, the Exo@DEX can be efficiently internalized into macrophages and have a prominent inhibition on macrophages. Among the inflammation-related cells, macrophages play a dominant role in the trigger and process of AIH, which therefore provided valid evidence for the possibility of our study (Wang et al., 2018; Violatto et al., 2019). However, more specific and deeper interactions between macrophages, liver, and DEX-loaded Exos should be explored to further explain the mechanism of how it works.

Data from the above studies suggested that Exo@DEX administration has a higher therapeutic effect over conventional DEX therapy. Furthermore, Exo@DEX may have a broader application in liver other than Con A-induced AIH. Hopefully, Exo@DEX could be used in other immune-related diseases including uveitis by intravitreous injection. In conclusion, our study provides strong evidence for the possibility of Exos as a drug vehicle in the treatment of AIH and, hopefully, a wider range of other diseases.

## Data Availability Statement

The original contributions presented in the study are included in the article/Supplementary Material, further inquiries can be directed to the corresponding author/s.

## Ethics Statement

The Balb/c mice’s experiments were approved by the Institutional Animal Care and Use Committees at the Institute of Process Engineering, Chinese Academy of Sciences. The Balb/c mice were purchased from Vital River Laboratories (Beijing, China). The animals were maintained in accordance with the guidelines of Laboratory Animal Care. All the animals were acclimatized to the laboratory for at least 7 days before experiments.

## Author Contributions

YW and MS conceived the study. JZ, YL, and RJ collected and analyzed the data. JZ and YL wrote the manuscript. All authors revised the final manuscript.

## Conflict of Interest

The authors declare that the research was conducted in the absence of any commercial or financial relationships that could be construed as a potential conflict of interest. The reviewer YY declared a shared affiliation, with no collaboration, with one of the authors, JZ, to the handling editor at the time of the review.

## Supplementary Material

Supplementary Figure 1 The size distribution and zeta potential of Exo@DEX. Size distribution and zeta potential of Exo@DEX measured by NTA before and after freezing at −80°C.

Supplementary Figure 2 The original data of Figure 1G. Western blotting analysis of Exo and Exo@DEX by ProteinSimple Wes^TM^ capillary western blot analyzer.

Supplementary Figure 3 Characterization of MSCs. Flow cytometry analysis of MSC markers.

Supplementary Figure 4 HPLC analysis of DEX. HPLC analysis of the incorporation of DEX into Exos.

Supplementary Figure 5 Cellular internalization of Exo@DEX. Representative fluorescence images of Exo uptake by RAW264.7 macrophages and L02 liver cells *in vitro* (images were enlarged, indicated by a white dotted box). Cells were incubated with Exo-free supernatant (Supernatant) or DiD-labeled Exo@DEX (Exo@DEX).

Supplementary Figure 6 Measurement of cytokines in supernatants. ELISA-based measurement of TNF-α, IFN-γ, and, IL-1β (pg/mL) in supernatants prepared from the culture medium after each different treatment (*n* = 5 for each group). Data are presented as the mean ± SD and were assessed via one-way ANOVA (*****P* < 0.0001).

Supplementary Figure 7 Location of *in vivo* EXO@DEX in macrophages. Presence of Exo@DEX in the liver specimen and their localization in FITC-labeled F4/80-positive macrophages after the injection of DiD-labeled Exo@DEX (white arrow) (red: DiD-labeled Exo@DEX, green: FITC-labeled F4/80 macrophages, and blue: DAPI-labeled cell nuclei).

Supplementary Figure 8 Levels of DEX measured in the liver. Mice were sacrificed 2 h after administration of DEX (0.5 mg/kg) or Exo@DEX (5 mg/kg). Levels of DEX in the liver were measured by HPLC. Data are presented as the mean ± SD and were assessed via two-tailed unpaired Student’s *t*-test (****P* < 0.001).

Supplementary Figure 9 Flow cytometry analysis of macrophage apoptosis in the liver. **(A)** Gating of F4/80-positive cells. **(B)** Flow cytometry analysis of annexin V-and-PI staining of F4/80-positive cells from single-cell suspension of the liver.
