# Supplementary figures and images for: Mesenchymal Stem Cells-Derived Exosomes as Dexamethasone Delivery Vehicles for Autoimmune Hepatitis Therapy
Source: Front Bioeng Biotechnol. 2021 Mar 30;9:650376. doi: 10.3389/fbioe.2021.650376 (PMC8042336; doi:10.3389/fbioe.2021.650376)

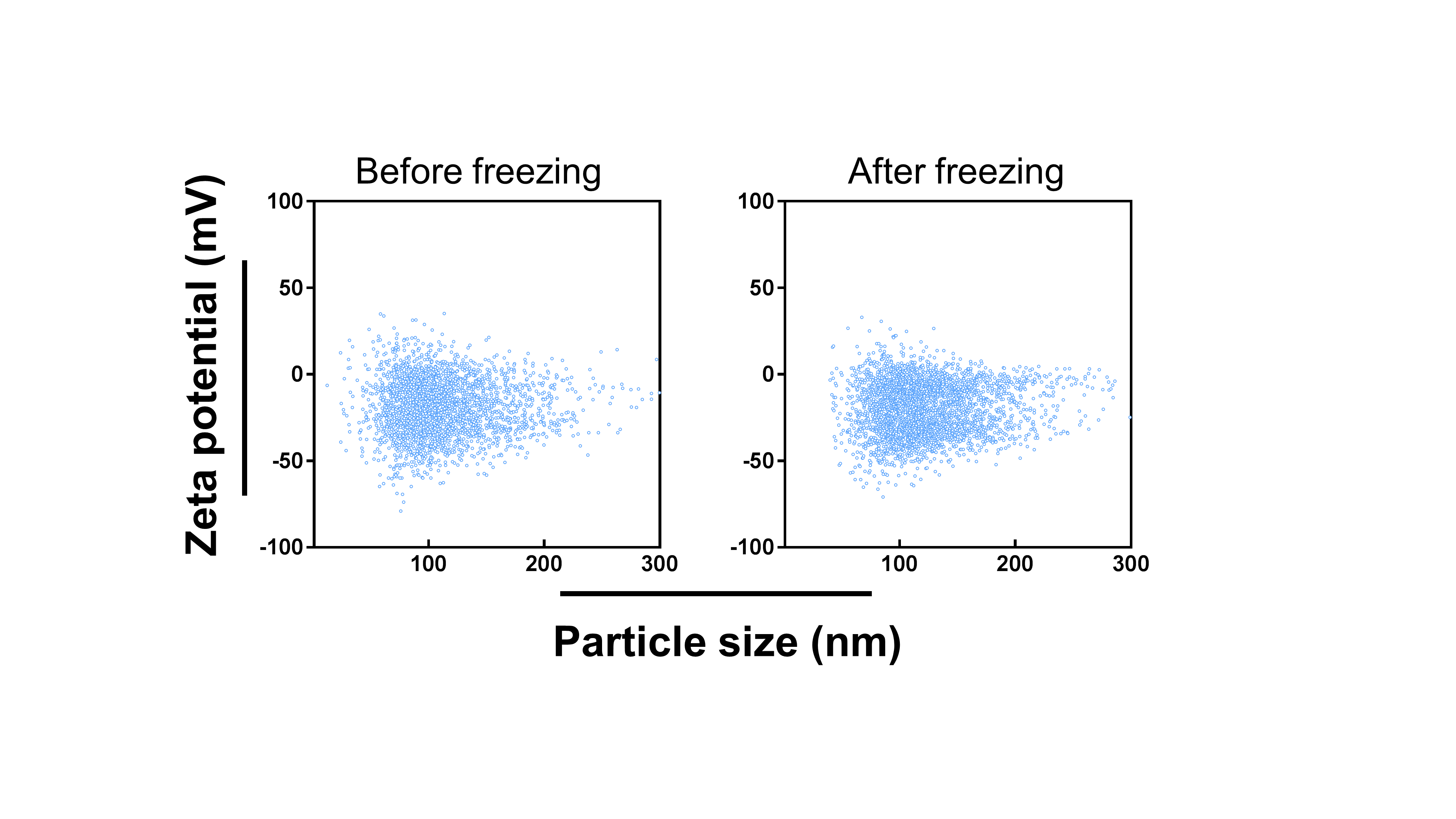

Supplement: Supplementary Figure 1 — The size distribution and zeta potential of Exo@DEX. Size distribution and zeta potential of Exo@DEX measured by NTA before and after freezing at −80°C. [file Image_1.TIF]

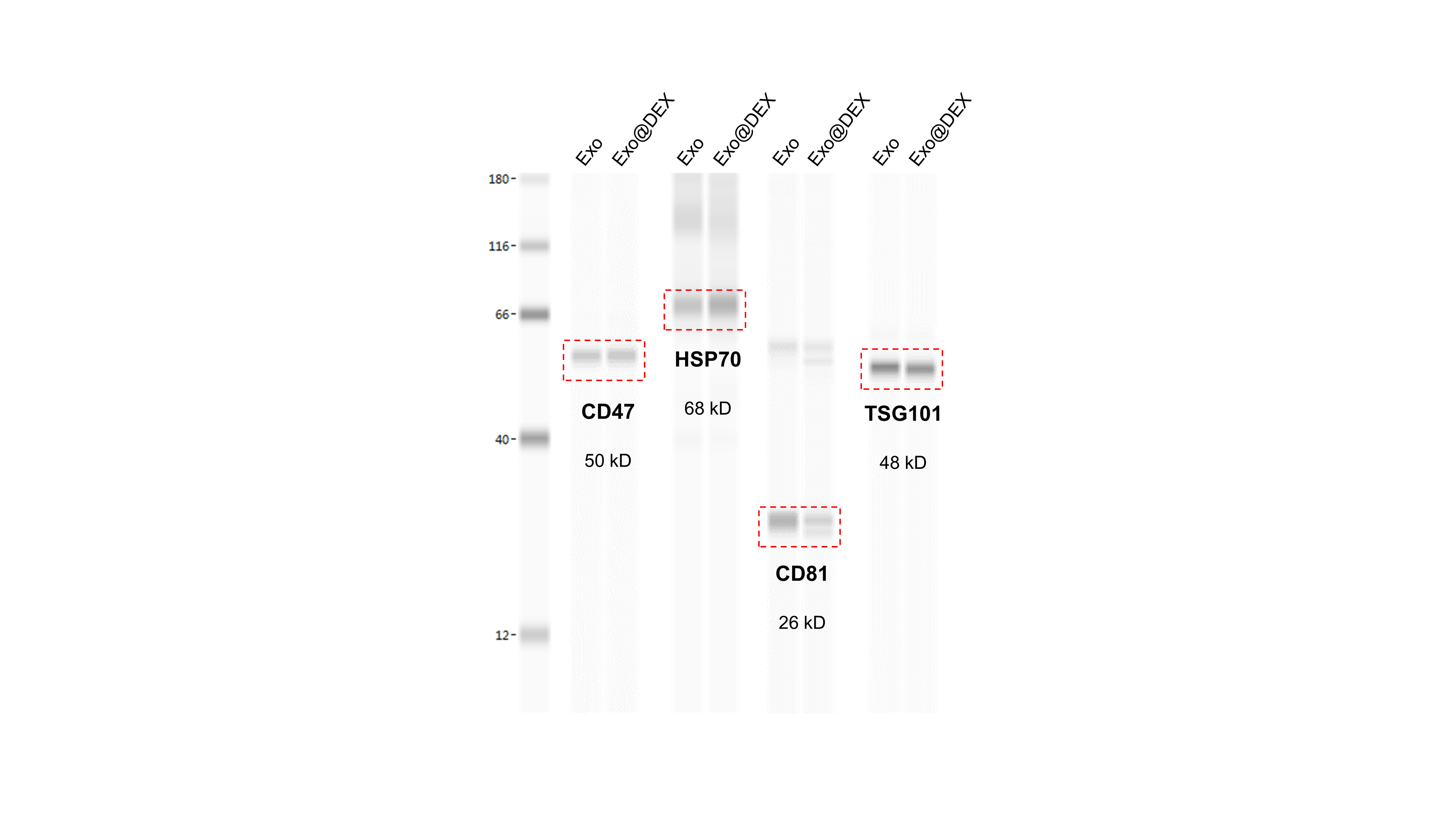

Supplement: Supplementary Figure 2 — The original data of Figure 1G. Western blotting analysis of Exo and Exo@DEX by ProteinSimple WesTM capillary western blot analyzer. [file Image_2.TIF]

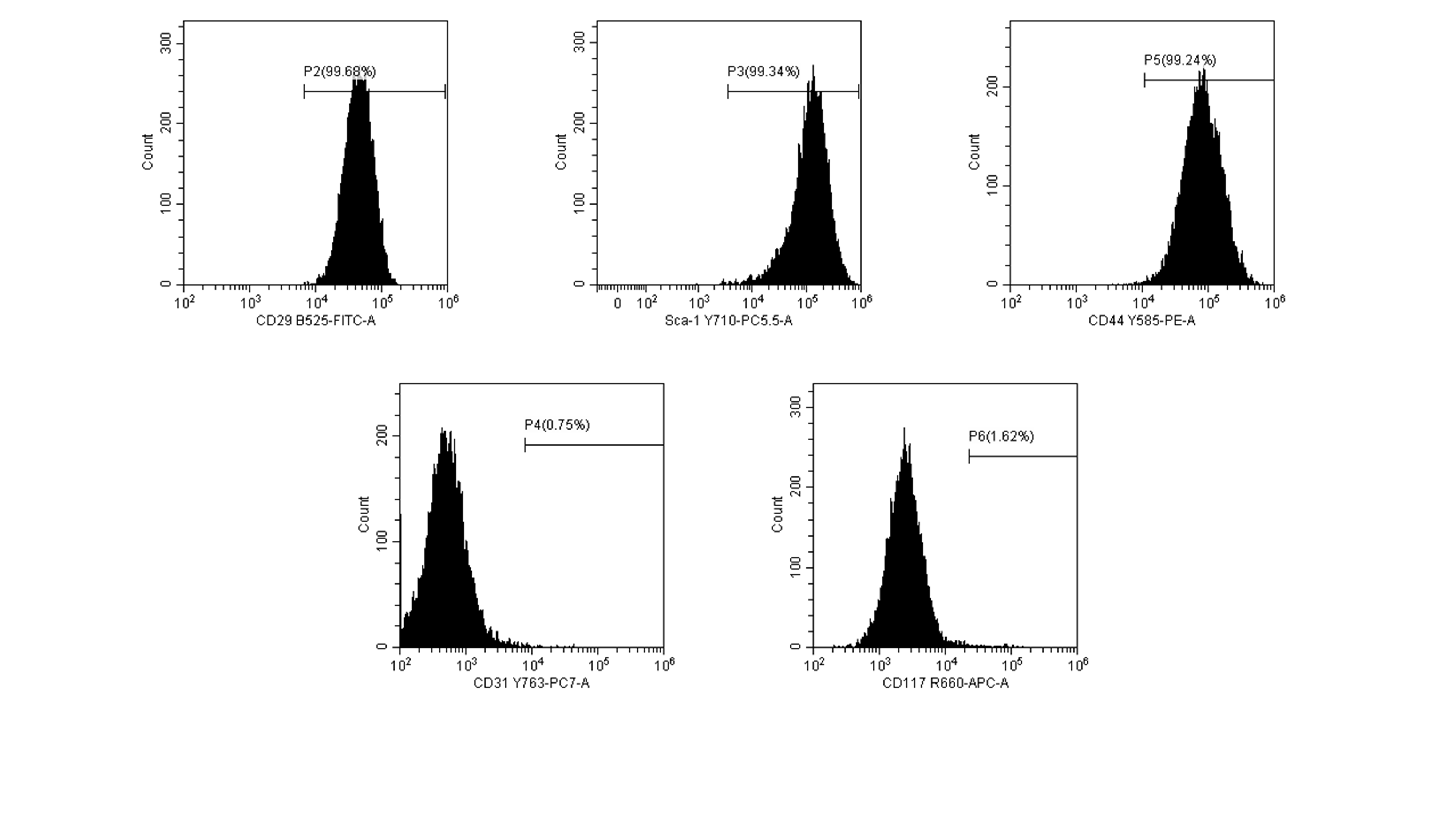

Supplement: Supplementary Figure 3 — Characterization of MSCs. Flow cytometry analysis of MSC markers. [file Image_3.TIF]

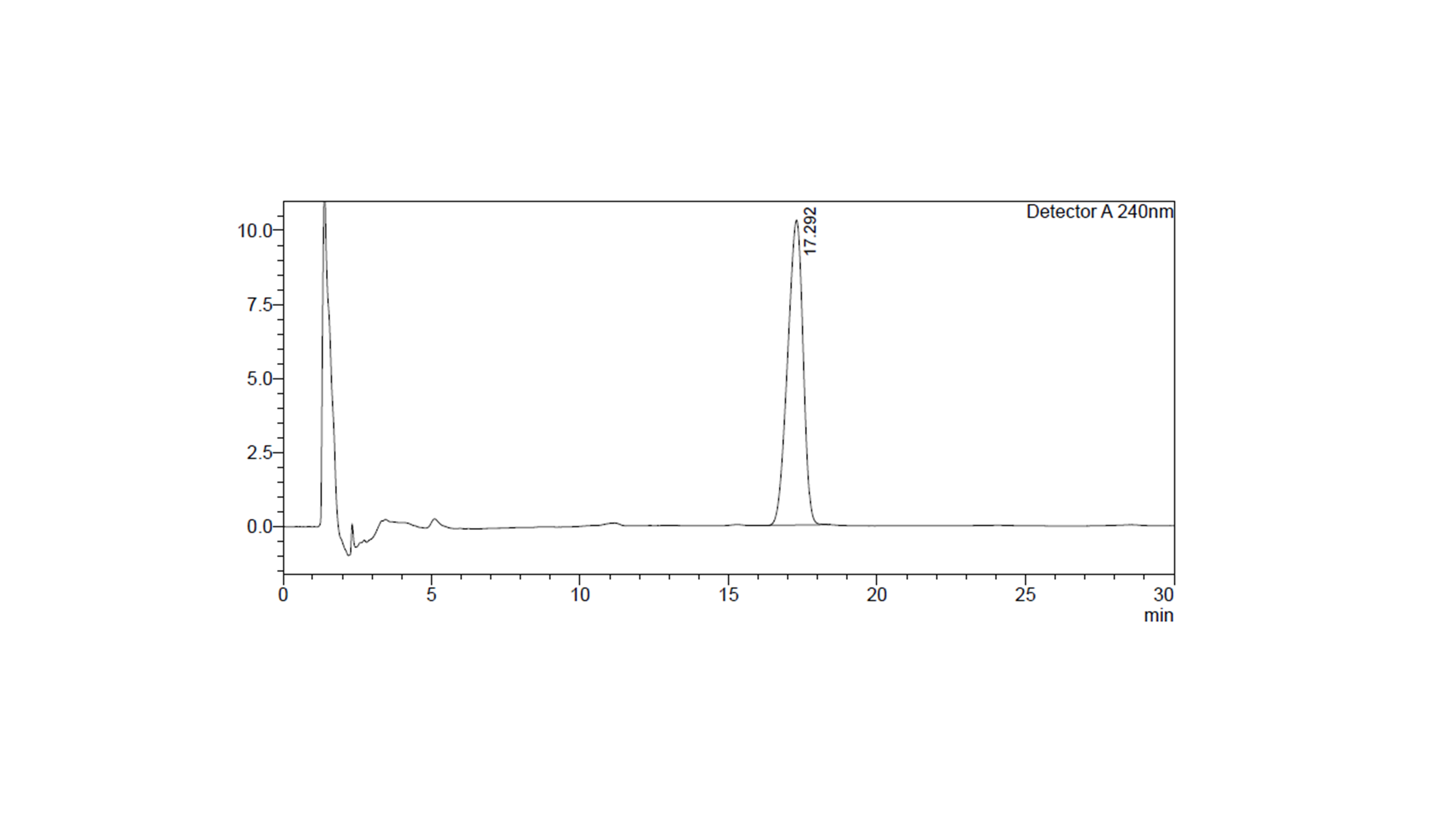

Supplement: Supplementary Figure 4 — HPLC analysis of DEX. HPLC analysis of the incorporation of DEX into Exos. [file Image_4.TIF]

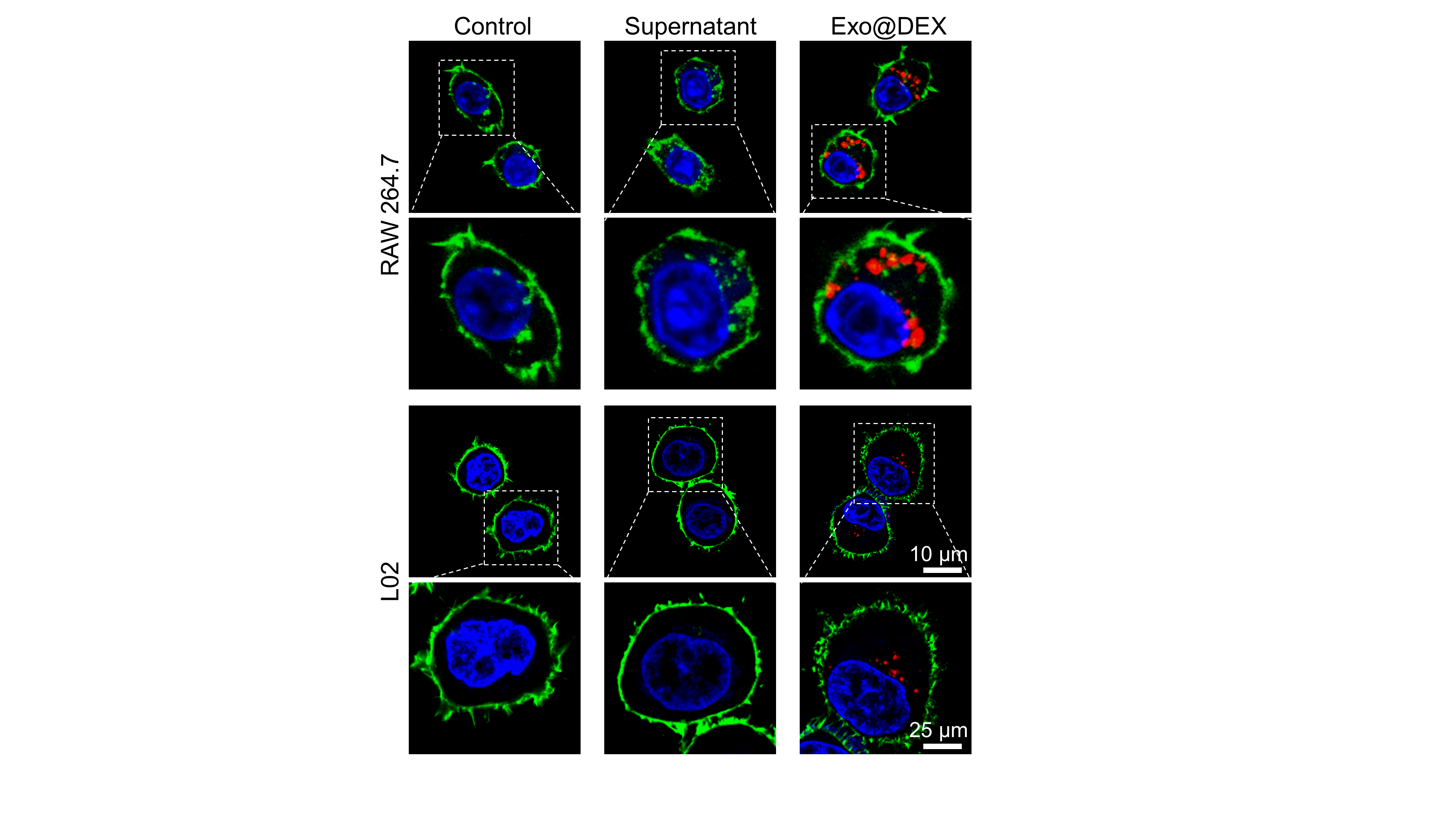

Supplement: Supplementary Figure 5 — Cellular internalization of Exo@DEX. Representative fluorescence images of Exo uptake by RAW264.7 macrophages and L02 liver cells in vitro (images were enlarged, indicated by a white dotted box). Cells were incubated with Exo-free supernatant (Supernatant) or DiD-labeled Exo@DEX (Exo@DEX). [file Image_5.TIF]

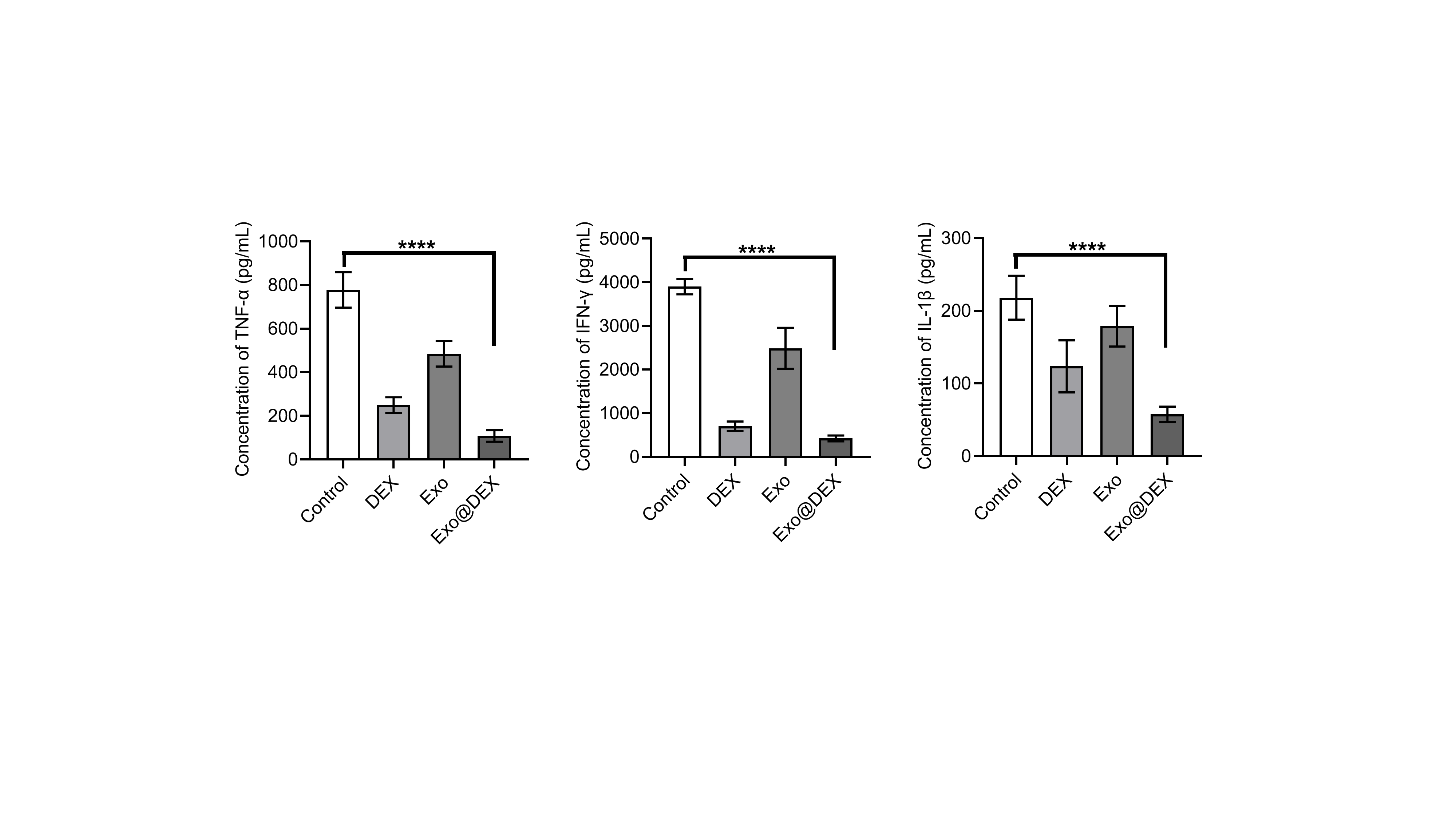

Supplement: Supplementary Figure 6 — Measurement of cytokines in supernatants. ELISA-based measurement of TNF-α, IFN-γ, and, IL-1β (pg/mL) in supernatants prepared from the culture medium after each different treatment (n = 5 for each group). Data are presented as the mean ± SD and were assessed via one-way ANOVA (****P < 0.0001). [file Image_6.TIF]

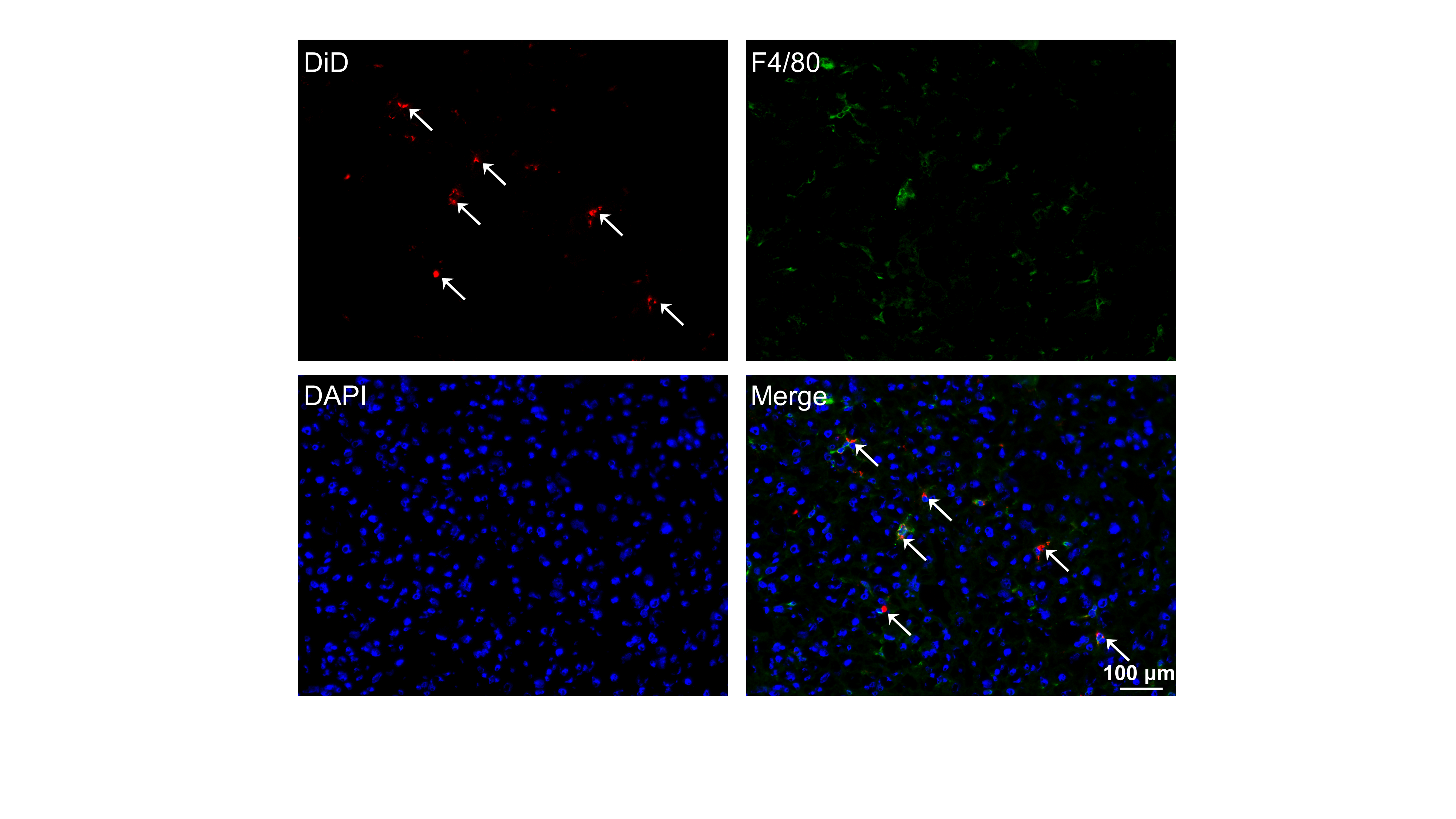

Supplement: Supplementary Figure 7 — Location of in vivo EXO@DEX in macrophages. Presence of Exo@DEX in the liver specimen and their localization in FITC-labeled F4/80-positive macrophages after the injection of DiD-labeled Exo@DEX (white arrow) (red: DiD-labeled Exo@DEX, green: FITC-labeled F4/80 macrophages, and blue: DAPI-labeled cell nuclei). [file Image_7.TIF]

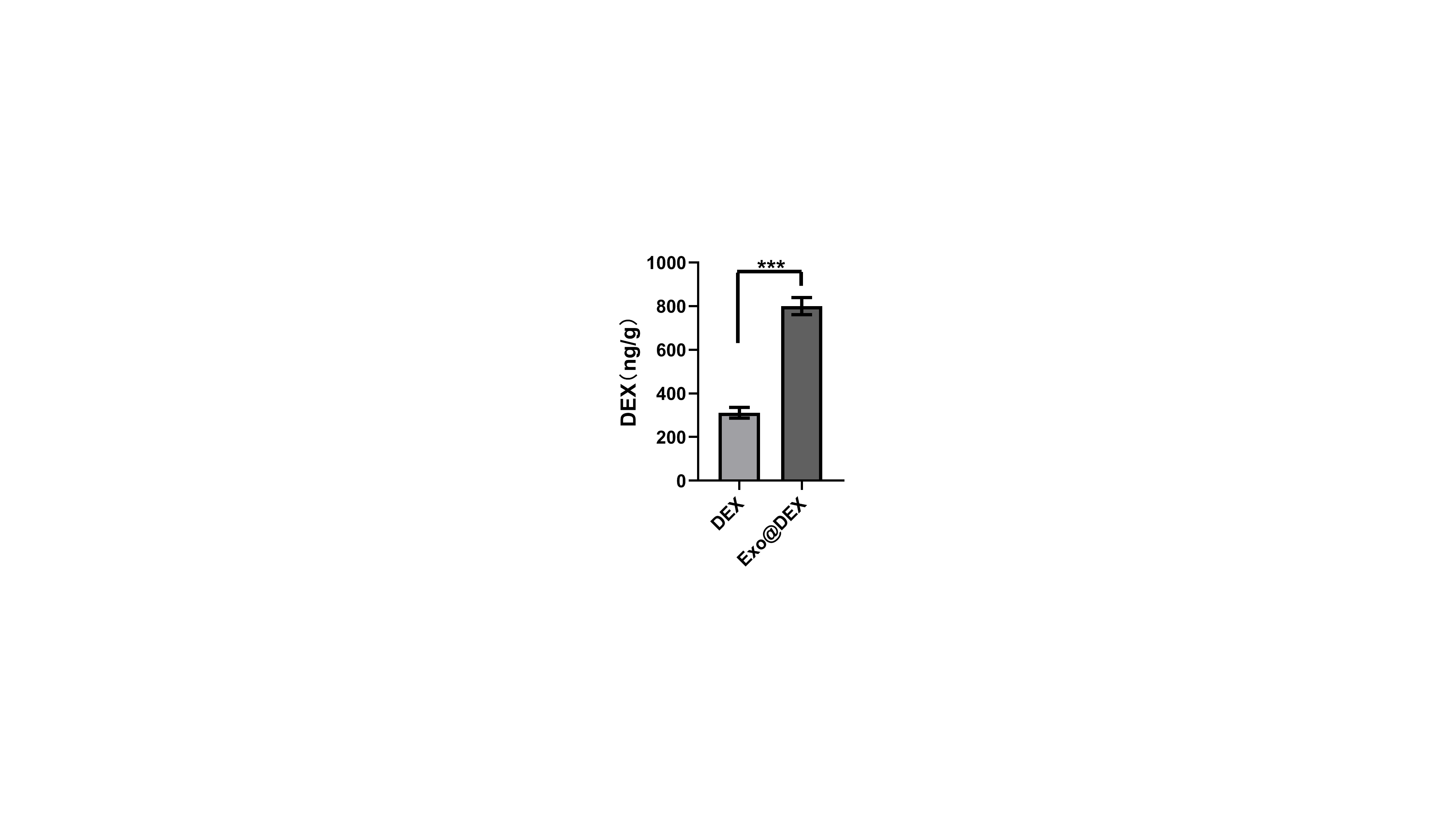

Supplement: Supplementary Figure 8 — Levels of DEX measured in the liver. Mice were sacrificed 2 h after administration of DEX (0.5 mg/kg) or Exo@DEX (5 mg/kg). Levels of DEX in the liver were measured by HPLC. Data are presented as the mean ± SD and were assessed via two-tailed unpaired Student’s t-test (***P < 0.001). [file Image_8.TIF]

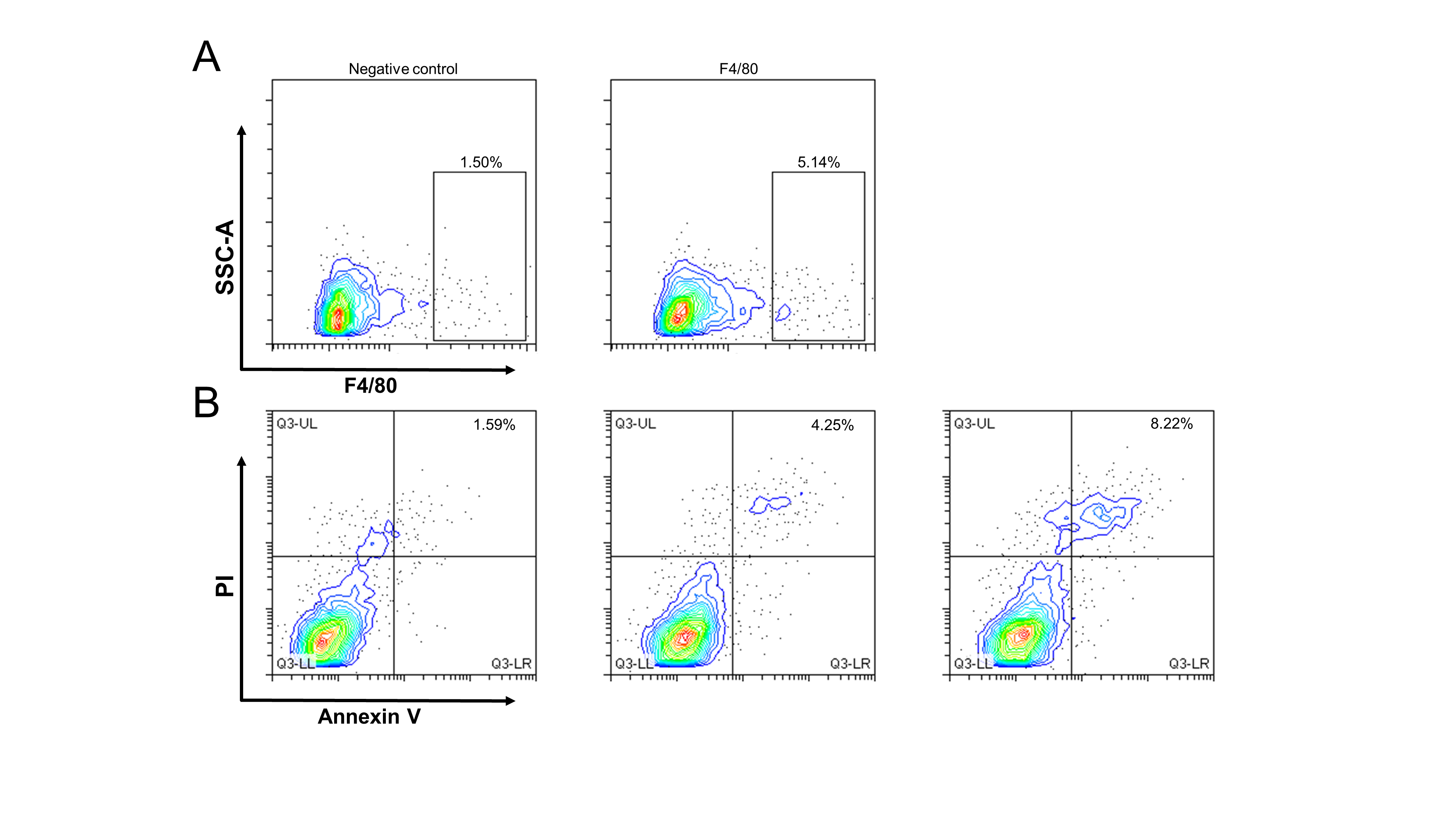

Supplement: Supplementary Figure 9 — Flow cytometry analysis of macrophage apoptosis in the liver. (A) Gating of F4/80-positive cells. (B) Flow cytometry analysis of annexin V-and-PI staining of F4/80-positive cells from single-cell suspension of the liver. [file Image_9.TIF]
